# Supplementary material for: A Simple Expression for the Screening of Excitonic Couplings between Chlorophylls as Inferred for Photosystem I Trimers
Source: Int J Mol Sci. 2024 Aug 19;25(16):9006. doi: 10.3390/ijms25169006 (PMC11355009; doi:10.3390/ijms25169006)
Supplement: Supplementary file 1 [file ijms-25-09006-s001.zip › si.pdf]

# Supplementary Materials: A simple expression for the screening of excitonic couplings between chlorophylls as inferred for photosystem I trimers

**Matthias Eder[1] and Thomas Renger [1]***

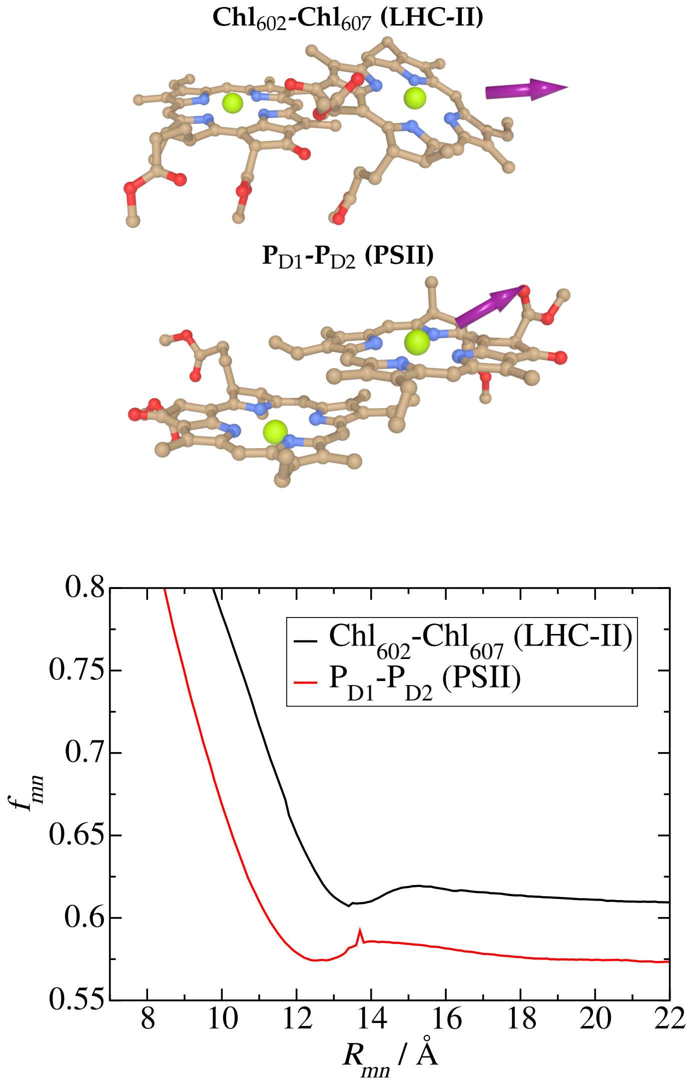


**Figure S1. Upper part:** Structure of $Chl_{602}$-$Chl_{607}$ dimer of light-harvesting complex LHC-II. **Middle part:** Structure of $P_{D1}$-$P_{D2}$ dimer of photosystem II. **Lower part:** Screening factor for $Chl_{602}$-$Chl_{607}$ (black line) and $P_{D1}$-$P_{D2}$ (red line) dimer as a function of interpigment distance, obtained with Poisson-TrEsp method (see main text). The distance was varied by displacing the pigments along the center-to-center direction, as illustrated by the purple arrows in the upper and middle panels.

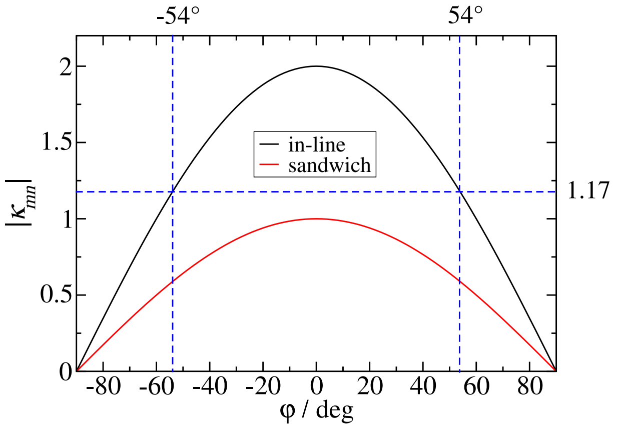


**Figure S2.** Absolute magnitude of orientational factor $\kappa_{mn}$ as function of rotation angle $\varphi$ for the model dimers in in-line (black line) and sandwich (red line) geometry of Figure 7 of the main text. The horizontal blue-dashed line marks the value $\kappa_0 = 1.17$, which is used in the empirical screening function $f_{mn}^{(4)}$ to switch from a constant screening value to an exponential distance dependence. The vertical blue-dashed lines provide the respective rotation angles for the in-line dimer.

**Table S1.** Same as in Table 3 of the main text but obtained in the many-cavity model.

| | $\sigma^2$ / cm$^{-2}$ | | |
|---|---|---|---|
| group | $f_{mn}^{(2)}$ | $f_{mn}^{(3)}(R_{mn})$ | $f_{mn}^{(4)}(R_{mn}, \kappa_{mn})$ |
| 1 | **1.13** | **1.84** | **1.14** |
| 2 | **2.47** | 10.98 | **2.37** |
| 3 | 16.95 | **3.86** | **3.35** |
| all | 4.03 | 5.95 | **1.98** |
